# Liver glucose metabolism in humans

**DOI:** 10.1042/BSR20160385

**Published:** 2016-11-29

**Authors:** María M. Adeva-Andany, Noemi Pérez-Felpete, Carlos Fernández-Fernández, Cristóbal Donapetry-García, Cristina Pazos-García

**Affiliations:** * Nephrology Division, Hospital General Juan Cardona, c/ Pardo Bazán s/n, 15406 Ferrol, Spain

**Keywords:** diabetes, glucokinase, glucose, hexosamine pathway, pentose phosphate pathway

## Abstract

Information about normal hepatic glucose metabolism may help to understand pathogenic mechanisms underlying obesity and diabetes mellitus. In addition, liver glucose metabolism is involved in glycosylation reactions and connected with fatty acid metabolism. The liver receives dietary carbohydrates directly from the intestine via the portal vein. Glucokinase phosphorylates glucose to glucose 6-phosphate inside the hepatocyte, ensuring that an adequate flow of glucose enters the cell to be metabolized. Glucose 6-phosphate may proceed to several metabolic pathways. During the post-prandial period, most glucose 6-phosphate is used to synthesize glycogen via the formation of glucose 1-phosphate and UDP–glucose. Minor amounts of UDP–glucose are used to form UDP–glucuronate and UDP–galactose, which are donors of monosaccharide units used in glycosylation. A second pathway of glucose 6-phosphate metabolism is the formation of fructose 6-phosphate, which may either start the hexosamine pathway to produce UDP-*N*-acetylglucosamine or follow the glycolytic pathway to generate pyruvate and then acetyl-CoA. Acetyl-CoA may enter the tricarboxylic acid (TCA) cycle to be oxidized or may be exported to the cytosol to synthesize fatty acids, when excess glucose is present within the hepatocyte. Finally, glucose 6-phosphate may produce NADPH and ribose 5-phosphate through the pentose phosphate pathway. Glucose metabolism supplies intermediates for glycosylation, a post-translational modification of proteins and lipids that modulates their activity. Congenital deficiency of phosphoglucomutase (PGM)-1 and PGM-3 is associated with impaired glycosylation. In addition to metabolize carbohydrates, the liver produces glucose to be used by other tissues, from glycogen breakdown or from *de novo* synthesis using primarily lactate and alanine (gluconeogenesis).

## INTRODUCTION

Understanding pathways of glucose metabolism in the liver of healthy humans may help to clarify metabolic alterations that occur in obesity and diabetes mellitus, two common diseases. In addition, glucose metabolism may be involved in other disorders as well, as glucose provides reducing equivalents for fatty acid synthesis, ribose 5-phosphate for nucleotide synthesis and precursors for glycosylation reactions. Carbohydrates derived from intestinal absorption are initially handled by hepatocytes whereas dietary fatty acids form triacylglycerols inside the enterocytes and reach the lymph stream assembled as chylomicrons that are incorporated to the bloodstream and finally arrive in the liver as remnant chylomicrons. In healthy individuals, the liver is a major site of glucose utilization during the post-prandial period, although hepatic contribution to glucose consumption relative to peripheral tissues has been found variable in different studies, from one-third to 50–60% of the glucose ingested. Peripheral glucose uptake including skeletal muscle and non-insulin sensitive tissues (predominantly the brain) accounts for the rest of total glucose disposal after meals [1]. To be utilized, glucose enters the hepatocyte and is phosphorylated to glucose 6-phosphate. Glucose 6-phosphate may follow a number of metabolic pathways, including glycogen synthesis, the hexosamine pathway, the pentose phosphate pathway and oxidative routes. Excess glucose is used to synthesize fatty acids in the liver. In addition to glucose utilization, human liver releases glucose to the systemic circulation, either from previously stored glycogen (glycogenolysis) or by generating glucose from precursors such as alanine, lactate and glycerol (gluconeogenesis). This unique ability of the human liver to store and release glucose is crucial to endure periods of fasting.

## HEPATIC GLUCOSE UTILIZATION

### Glucose transport inside the hepatocyte

Glucose entry into human hepatocytes is thought to be accomplished via glucose transporters that operate a passive (energy-independent) transport of glucose and it is usually accepted that glucose transporter-2 (solute carrier family 2, member A2, SLC2A2 or GLUT2) is the predominant hepatic glucose transporter in humans. GLUT2 is a facilitative glucose transporter that allows bidirectional fluxes of glucose in and out the cells. GLUT2 mRNA is expressed in human liver, kidney, pancreatic β-cells and small intestine (jejunum) [2,3]. Subcellular location of GLUT2 in human hepatocytes has not been reported. The transporter is likely expressed at the basolateral membrane of intestinal and renal epithelial cells, where it works together with the sodium-glucose co-transporter located to the apical membrane to facilitate the absorption of glucose from the intestinal lumen or from the kidney tubular fluid into the blood [3]. Glucose uptake by the liver is not affected by insulin, being hyperglycaemia rather than hyperinsulinaemia the primary determinant of hepatic glucose transportation inside the hepatocytes [4].

The human gene encoding GLUT2 (*SLC2A2*) has been mapped to chromosome 3q26.1-q26.3 [2] and its organization and partial sequence have been reported. The putative GLUT2 sequence has three possible sites for asparagine-linked glycosylation that may be important in GLUT2 regulation [3]. The transcription of the *SLC2A2* gene and therefore the expression of GLUT2 is regulated by some hepatocyte nuclear factors (HNFs), including HNF4α, HNF1α and HNF1β [5–7].

In the liver, GLUT2 facilitates glucose release to the bloodstream whereas in the pancreas GLUT2 contributes to glucose-mediated insulin secretion. Mutations in the *SLC2A2* gene may cause Fanconi–Bickel disease and neonatal diabetes mellitus due to deficient insulin secretion. Likewise, mutations in genes encoding HNFs that reduce GLUT2 expression cause similar clinical phenotypes.

Fanconi–Bickel disease (glycogen storage disease type XI) is an autosomal recessive disorder caused by biallelic inactivating mutations in *SLC2A2*. Clinical manifestations include stunted growth and short stature, ketotic hypoglycaemia during periods of fasting, hepatomegaly secondary to glycogen accumulation, post-prandial hyperglycaemia and hypergalactosaemia, and kidney involvement characterized by glycogen accumulation in the proximal tubule, galactosuria, glucosuria in the presence of normal or low plasma glucose concentration, hyperaminoaciduria and hyperphosphaturia that may cause hypophosphataemic rickets. Fanconi–Bickel syndrome has been detected via neonatal screening for galactosaemia. Some patients present with a very mild phenotype [8]. Marked urinary bicarbonate loss contributing to metabolic acidosis has been observed in some patients. Severe ketosis and ketonuria with normal glycaemia has been reported in a patient with Fanconi–Bickel disease [9].

Neonatal diabetes, sometimes presenting as diabetic ketoacidosis, may be the initial clinical manifestation of mutations in the *SLC2A2* gene. Homozygous GLUT2 mutations are identified in approximately 5% of patients with neonatal diabetes mellitus, either transient or permanent. These patients develop Fanconi–Bickel syndrome later in life. The findings that patients with homozygous *SLC2A2* mutations may present with neonatal diabetes suggest that GLUT2 expressed in pancreatic β-cells is involved in glucose-stimulated insulin secretion [10].

Mutations in genes coding some HNFs produce maturity-onset diabetes of youth (MODY), a group of autosomal dominant forms of diabetes mellitus characterized by impaired glucose-stimulated insulin secretion and normal sensitivity to insulin, suggesting that pancreatic β-cell dysfunction rather than insulin resistance is the primary defect [5].

### Glucose phosphorylation to glucose 6-phosphate in the liver: glucokinase

Within the cells, free glucose is phosphorylated by hexokinase isoenzymes to yield glucose 6-phosphate. Four isoenzymes of hexokinase (I, II, III and IV or glucokinase) are known to exist in human tissues, but little is known about their distribution and differential function. Unlike other hexokinases, human glucokinase is not inhibited by its product, glucose 6-phosphate, and has lower molecular mass [11,12]. Glucokinase phosphorylates the sixth carbon of glucose at the expense of ATP. The crystal structure of human glucokinase has been solved, revealing that it is a monomeric enzyme with a single active site [13]. The Michaelis–Menten constant (*K*_m_) of human glucokinase for D-glucose is relatively high and approximates the physiological concentration of plasma glucose, 5 mM. The affinity of glucokinase for glucose is relatively low compared with other hexokinases. Human glucokinase has a sigmoidal saturation curve for glucose whereas other hexokinases have Michaelis–Menten hyperbolic kinetics. The inflexion point on the sigmoidal glucose saturation curve is at 8 mM [13].

Glucokinase is present in human pancreatic islet tissue but it is not found in the exocrine pancreas. In cultured hepatocytes obtained from human liver biopsies, glucokinase accounts for 95% of the glucose phosphorylation activity [11].

Recombinant pancreatic and liver human glucokinase undergo post-translational modification by small ubiquitin-like modifier (SUMO) proteins, but the physiological relevance of this finding is unclear [14].

*In vitro* studies suggest that transcriptional control of human glucokinase is carried out by insulin and glucagon. In cultured hepatocytes obtained from human liver biopsy samples, insulin increases glucokinase mRNA and enhances glucokinase activity whereas glucagon has the opposite effect [11].

In the human liver, glucokinase action is inhibited by glucokinase regulatory protein that acts as a competitive inhibitor of glucose binding to glucokinase. The structure of full-length cDNA for human glucokinase regulatory protein has been described. The gene encoding the glucokinase regulatory protein, glucokinase regulator (*GCKR*), maps to chromosome 2p23. The glucokinase regulatory protein is expressed in human liver and at very low levels in human islets and adipocytes (a tenth of that seen in liver) [13].

Fructose 1-phosphate activates glucokinase whereas fructose 6-phosphate suppresses glucokinase activity by acting on the glucokinase regulatory protein [15]. Fructose metabolism in humans is thought to occur predominantly in the liver. Fructose taken up by hepatocytes may be converted into either fructose 6-phosphate by hexokinase isoenzymes or fructose 1-phosphate by fructokinase [16]. The two initial products of hepatic fructose metabolism have opposite effects on the glucokinase regulatory protein. Glucokinase activators have been considered as a therapy for diabetes mellitus with inconclusive results. The inhibitory effect of fructose 1-phosphate on the glucokinase regulatory protein may be important to activate glucokinase and promote hepatic glucose metabolism.

The single gene encoding human glucokinase (*GCK*) is located on chromosome 7p. This gene contains two tissue-specific promoters, one active in pancreatic β-cells and the other active in liver, that generate two different isoenzymes of glucokinase in human tissues, glucokinase-1 in pancreatic β-cells and glucokinase-2 in hepatocytes. The tissue-specific promoters allow the glucokinase gene to be independently regulated in liver and pancreatic β-cells. Comparison of the nucleotide sequences of the human islet glucokinase cDNA with that of the human liver glucokinase cDNA reveals that the two cDNAs differ on their 5′-ends while having identical 3′-ends. These nucleotide sequences predict proteins that differ by 15 NH_2_-terminal residues [17]. Isoenzymes of glucokinase catalyse the initial step in the utilization of glucose by the pancreatic β-cells and hepatocytes, being activated by glucose binding to produce glucose 6-phosphate. In the pancreatic β-cells, the activity of glucokinase-1 leads to glucose-stimulated insulin secretion whereas in the hepatocyte glucokinase-2 promotes glucose uptake and utilization mainly via glycogen synthesis by maintaining a gradient for glucose transport into these cells [17].

Mutations in the glucokinase gene lead to disturbances in glucose metabolism, highlighting the crucial effect of the enzyme. Heterozygous inactivating (loss of function) mutations in the *GCK* locus result in MODY2 or GK-MODY, homozygous inactivating mutations produce permanent neonatal diabetes mellitus, and heterozygous activating (gain of function) *GCK* mutations cause autosomal dominant hyperinsulinism (persistent hyperinsulinaemic hypoglycaemia of infancy).

Heterozygous inactivating mutations in the human *GCK* gene cause MODY2, an autosomal dominant form of monogenic diabetes, also called familial mild fasting hyperglycaemia. In 1992, genetic linkage between MODY2 and the *GCK* gene was reported and the identification of a nonsense mutation in this gene was linked to MODY2 in one family [18]. Patients with MODY2 suffer impaired glucose-stimulated insulin secretion and alterations in hepatic glucose metabolism. Reduced insulin secretion by pancreatic β-cells is attributed to the decreased rate of glucokinase activity in these cells. In addition, glucokinase-deficient patients have decreased net accumulation of hepatic glycogen and relatively augmented hepatic gluconeogenesis after meals. Glucokinase deficiency affects the flux of the direct pathway of glycogen synthesis, which is maintained via gluconeogenesis [19].

Homozygous inactivating mutations in the glucokinase gene produce permanent neonatal diabetes mellitus, a rare autosomal recessive disorder with a profound defect in β-cell function [20]. Heterozygous activating mutations in the *GCK* gene may cause autosomal dominant hyperinsulinism. Patients suffer from non-ketotic hypoglycaemic episodes associated with inappropriately high serum levels of insulin. When expressed *in vitro*, the mutant enzyme has increased affinity for glucose that results in higher rate of glucose metabolism and enhanced insulin secretion [21].

### Glucose 6-phosphate metabolism

**Figure 1:**
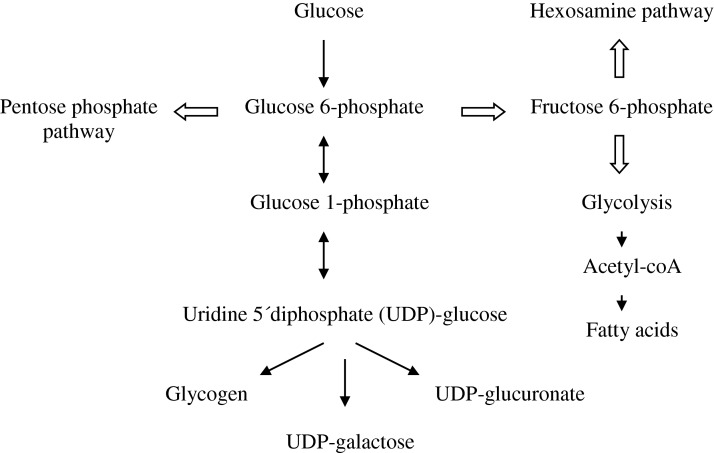
Summary of glucose utilization in the human liver

Glucose 6-phosphate may follow three known metabolic routes, namely isomerization into glucose 1-phosphate, isomerization into fructose 6-phosphate and oxidation into gluconolactone (Figure 1). Glucose 1-phosphate is transformed into UDP–glucose, which is the precursor of glycogen, UDP–glucuronate and UDP–galactose. Fructose 6-phosphate may either start the hexosamine pathway by combining with glutamine or continue into the glycolytic pathway to form pyruvate and then acetyl-CoA. Oxidation of glucose 6-phosphate to gluconolactone starts the pentose phosphate pathway.

#### Formation of glucose 1-phosphate and UDP–glucose

##### Phosphoglucomutase-1: isomerization of glucose 6-phosphate and glucose 1-phosphate

Phosphoglucomutase(PGM)-1 is a phosphotransferase that catalyses the reversible transfer of phosphate between the 1- and 6- positions of α-D-glucose and therefore the isomerization of glucose 1-phosphate and glucose 6-phosphate. Human PGM-1 is expressed in liver and muscle, being a highly polymorphic protein [22]. The crystal structure of the wild-type enzyme has been reported [23].

**Figure 2:**
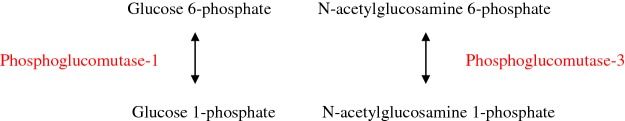
Phosphoglucomutase-1 and Phosphoglucomutase-3 reactions

There are four isoenzymes of human phosphoglucomutase: PGM1, PGM2, PGM3 and PGM5. The PGM isoenzyme of human milk attributed to a fourth locus, PGM4, shows similar cross-reactivity as PGM-1 suggesting close structural similarity. PGM-1 is an efficient phosphoglucomutase compared with PGM-2. By contrast, PGM-2 has a high affinity for ribose 1-phosphate, being an efficient phosphoribomutase [22]. PGM-3 has a low affinity for ribose 1-phosphate and is a poor phosphoglucomutase. Instead, this enzyme catalyses the reversible interconversion of *N*-acetylglucosamine 6-phosphate and *N*-acetylglucosamine 1-phosphate, being required for the synthesis of UDP-*N*-acetylglucosamine in the hexosamine pathway (Figure 2) [24]. Human *PGM1* gene has been mapped to 1p31 and its complete structure has been ascertained, discovering two alternatively spliced first exons. The human gene encoding PGM-3 (*AGM1*) maps to chromosome 6. Human *PGM5* gene maps to the centromeric region of chromosome 9, being an expressed gene with widespread distribution in human tissues [22].

In 2009, heterozygous mutations in the *PGM1* gene were identified in a 35-year-old man with intolerance to physical activity and exercise-induced episodes of rhabdomyolysis. A skeletal muscle biopsy revealed accumulation of normally structured glycogen suggesting impaired utilization of glycogen-derived glucose. PGM-1 activity in skeletal muscle was reduced to 1% of the value among controls. PGM-1 deficiency was proposed to be designated glycogen storage disease type XIV [25]. In a patient with PGM-1 deficiency, plasma glucose levels decline during submaximal exercise and glucose infusion reduces heart rate and ameliorates exercise perception, compared with control subjects. The patient tends to oxidize palmitate at higher rate than the control subjects. These findings suggest that deficiency of PGM-1 is associated with a failure to utilize glycogen as energy source both in the liver and in the skeletal muscle due to the isomerization defect that prevents the formation of glucose 6-phosphate from glycogen-derived glucose 1-phosphate [26].

In addition to this clinical phenotype, congenital deficiency of PGM-1 is associated with impaired protein glycosylation. In 2012, applying whole-exome sequencing in patients with unsolved congenital disorders of glycosylation (CDG), mutations in the *PGM1* gene were found to be the cause of a CDG named PGM1-CDG, revealing for the first time the crucial role of PGM-1 in glycosylation reactions [27].

Protein glycosylation involves the attachment of oligosaccharide structures to proteins via either N-linkage on asparagine or O-linkage on serine and threonine residues. The first step of N-glycosylation of proteins is the assembly of glycan precursors, each composed of a chain of monosaccharide units attached to dolichol in the membrane of the endoplasmic reticulum. Then, the glycan precursors are affixed to asparagine residues in the nascent peptide chain of a protein. The glycosylated peptide chain is transferred to the Golgi apparatus where the glycan precursors are modified to generate the mature glycoprotein. CDG type 1 are due to defects on the first part of the protein N-glycosylation pathway, either the dolichol-glycan assembly or the glycan transfer to the nascent protein in the endoplasmic reticulum. CDG type 2 are due to errors on the modification of the attached glycan precursor in the Golgi apparatus. Congenital deficiency of PGM-1 is associated with a mixed disorder of N-glycosylation and patients affected with this disease show features of both type 1 and type 2 congenital glycosylation disorders. Both protein folding defects and compromised catalysis of PGM-1 may play a role in the disease [27,28].

The clinical phenotype of patients with PGM-1 deficiency includes short stature, bifid uvula with or without cleft palate at birth, neurological symptoms such as psychomotor retardation and seizures, intolerance to exercise, dilated cardiomyopathy, episodes of ketotic hypoglycaemia, hepatopathy and coagulation abnormalities [27,28].

Supplementation with oral D-galactose (0.5–1 g/kg/day and maximum 50 g/day) has been proposed as therapy for PGM-1 deficiency, but a lactose-rich diet has not improved the clinical outcome in one patient with the disease [29].

The pathophysiological mechanisms connecting PGM-1 deficiency with CDG and the clinical phenotype of the disease are unclear. In patients with PGM-1 deficiency, fibroblasts supplemented with galactose show restoration of protein glycosylation, suggesting that the intermediates required for glycosylation reactions may be produced in the presence of galactose. In addition, there is no evidence of glycogen accumulation in PGM-1 deficient, galactose-supplemented fibroblasts, suggesting that glycogen may be used by these cells [28].

##### Formation of UDP–glucose: UDP–glucose pyrophosphorylase (glucose 1-phosphate uridyltransferase)

**Figure 3:**
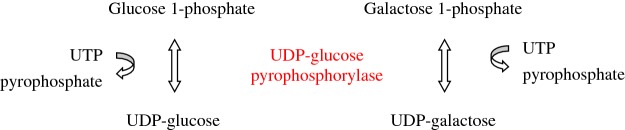
Glucose 1-phosphate uridyltransferase or uridine 5′diphosphate (UDP)-glucose pyrophosphorylase (UGP) reactions

The enzyme glucose 1-phosphate uridyltransferase or UDP–glucose pyrophosphorylase (UGP) catalyses the reversible formation of UDP–glucose and PP_i_ from glucose 1-phosphate and UTP in the presence of Mg^2+^. The crystal structure of the human UGP has been determined, revealing that the enzyme adopts an octameric structure [30]. In addition to UDP–glucose formation from glucose 1-phosphate, *in vitro* analyses reveal that human UGP may catalyse a similar reaction with galactose, the formation of UDP–galactose from galactose 1-phosphate and UTP (Figure 3) [31]. Two isoenzymes of UGP are known in humans, UGP1 and UGP2, encoded by different genes. The gene encoding UGP1 is located on chromosome 1q21-q23 whereas the gene coding for UGP2 has been assigned to chromosome 2p13-p14. Northern blotting analysis reveals that UGP1 gene is expressed at the highest level in skeletal muscle, followed by liver, heart and kidney. UGP2 has been isolated from skeletal muscle [32].

In the liver, UDP–glucose may be involved in several metabolic pathways, including glycogen synthesis, formation of UDP–glucuronate and formation of UDP–galactose. UDP–glucose is the immediate glucose donor for the synthesis of glycogen, supplying glucose residues for the initiation and the elongation of the glycogen particle. UDP–glucose is involved in the synthesis of UDP–glucuronate, which facilitates the excretion of endogenous compounds such as bilirubin and foreign molecules such as acetaminophen by converting them into more polar metabolites. In addition, UDP–glucose may be utilized to generate UDP–galactose from galactose 1-phosphate.

###### Glycogen Synthesis

In the human liver, a major quantitative pathway of glucose utilization is glycogen synthesis, glucose being stored as glycogen inside the hepatocytes until glycogen storage is filled. Food-derived glucose provides the direct pathway to synthesize glycogen after meals. Glucose derived from gluconeogenesis represents the indirect pathway and contributes to glycogen formation both during fed and fasted states. Approximately 50% of the glucose ingested is stored as glycogen during the post-prandial period in healthy subjects. After a meal, the majority of glycogen is formed via the direct pathway (73%), but the indirect pathway accounts for 27% of the glycogen formation [33]. The ability to store glucose as glycogen is reduced in patients with impaired glucose tolerance. Dietary carbohydrate overfeeding rises the concentration of liver glycogen in healthy volunteers. However, the capacity to store glycogen in the liver after carbohydrate overfeeding is limited and continued accumulation of liver glycogen, when the reserve is complete, is attenuated by an increase in glycogen release that occurs with increasing glycogen content [34]. Excess dietary glucose not stored as glycogen is converted into fat by hepatic *de novo* lipogenesis, which is an energetically expensive process [35].

To synthesize glycogen, glucose is phosphorylated upon its entrance into the hepatocytes. Glucose 6-phosphate is isomerized into glucose 1-phosphate by PGM-1 and then converted into UDP–glucose by UGP. UDP–glucose is the immediate glucose donor for glycogen synthesis. Glycogenin initiates the synthesis of glycogen by autoglycosylation transporting glucose residues from UDP–glucose to itself, forming a short linear chain of approximately 10–20 glucose moieties. The elongation of this initial glycogen sequence is accomplished by glycogen synthase that transfers glucose moieties from UDP–glucose to the growing glycogen strand, providing the linear linkages between glucose residues. Glycogen branching enzyme introduces branch points in the glycogen particle. There are two isoforms of human glycogenin, glycogenin-1 and glycogenin-2, encoded by *GYG1* and *GYG2* genes respectively. Both isoenzymes are present in human liver. Diabetic males harbouring a *GYG2* deletion lack glycogenin-2 in liver biopsy samples, but they are able to synthesize glycogen, likely because they show glycogenin-1 expression in the liver [36]. There are two isoenzymes of glycogen synthase in humans, glycogen synthase-1 (GYS1), which is the isoform present in skeletal muscle and heart, and glycogen synthase-2 (GYS2), the liver isoenzyme. Congenital deficiency of liver glycogen synthetase (GYS2) due to mutations in the *GYS2* gene on chromosome 12p12.2 is an autosomal recessive disease named glycogen storage disease type 0. Defective glycogen synthesis after meals leads to post-prandial hyperglycaemia, glucosuria and hyperlactatemia. Ketotic hypoglycaemia and ketonuria develop during fasting periods owing to low liver glycogen content. Diagnosis of the disease may require frequent measurements of blood glucose, lactate and ketone bodies in both the fed and fasting states (24-h metabolic profile), which show the characteristic biochemical disturbances. In liver biopsy samples, hepatocytes contain small amounts of glycogen and show moderate steatosis [37]. Congenital deficiency of glycogen branching enzyme is an autosomal recessive disorder that leads to accumulation of abnormally structured glycogen with fewer branched points resembling amylopectin in multiple tissues, including liver, heart, skeletal muscle and the nervous system.

###### Formation of UDP–glucuronate: UDP–glucose dehydrogenase

In the human liver, a minor amount of UDP–glucose is converted to UDP–glucuronate that yields glucuronate residues to a variety of exogenous and endogenous compounds to allow their solubilization and excretion. Glucuronate residues are also incorporated to nascent glycosaminoglycans such as hyaluronan. The enzyme UDP–glucose dehydrogenase encoded by the gene *UGDH* converts UDP–glucose into UDP–glucuronate through two successive NAD^+^-dependent oxidation steps [38]. Human UDP–glucose dehydrogenase apoprotein has been purified [39]. Glucuronate residues derived from UDP–glucuronate are attached to endogenous molecules such as bilirubin and foreign compounds such as acetaminophen to assist in their elimination. The enzymes that attach glucuronate residues to a variety of chemical compounds are isoenzymes of UDP–glucuronosyltransferase [38].

###### Formation of UDP-galactose: galactose 1-phosphate uridyltransferase

**Figure 4:**
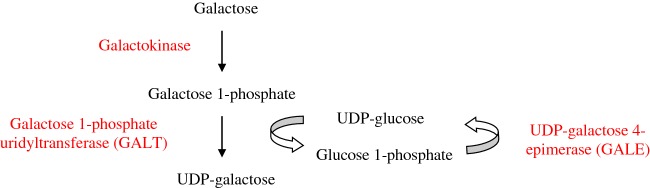
Galactose metabolism

Dietary β-D-galactose is metabolized by the Leloir pathway, composed of four enzymatic steps (Figure 4). First, β-D-galactose is epimerized to α-D-galactose by galactose mutarotase. Second, α-D-galactose is phosphorylated to galactose 1-phosphate by galactokinase. Third, the reaction galactose 1-phosphate + UDP–glucose generates UDP–galactose + glucose 1-phosphate. This reaction is catalysed by galactose 1-phosphate uridyltransferase (GALT) that transfers an UMP group from UDP–glucose to galactose 1-phosphate thereby generating UDP–galactose and glucose 1-phosphate. Finally, UDP–galactose is epimerized to UDP–glucose by UDP–galactose 4-epimerase (GALE). The X-ray crystallographic structure of this enzyme has been reported [40].

In humans, defects in the genes encoding galactokinase, GALT or GALE cause galactosaemia. Congenital defects in GALT result in galactosaemia-1 or classic galactosaemia, congenital deficit of galactokinase causes galactosaemia-2 whereas GALE deficiency leads to a rare form of galactosaemia. No human disease has been associated with galactose mutarotase deficiency, although the human gene encoding this protein has been cloned.

In patients with classic galactosaemia due to GALT deficiency, galactose 1-phosphate is accumulated and the production of UDP–galactose is reduced, impairing the incorporation of galactose to proteins and lipids. A deficiency of galactose-containing glycoproteins and glycolipids is observed in these patients [31]. In addition to GALT action, UDP–galactose may be generated from galactose 1-phosphate by the enzyme UGP, as mentioned above (Figure 3). The ability of human UGP to convert galactose 1-phosphate into UDP–galactose may be important to alleviate galactose 1-phosphate accumulation in patients with classic galactosaemia due to GALT deficiency [31].

#### Isomerization of glucose 6-phosphate into fructose 6-phosphate: glucose 6-phosphate isomerase or phosphoglucoisomerase

Glucose 6-phosphate isomerase (GPI) is the enzyme that catalyses the reversible isomerization of fructose 6-phosphate and glucose 6-phosphate. The gene encoding GPI is located on chromosome 19q and its structure has been determined. Congenital GPI deficiency is a common disease leading to hydrops fetalis, chronic non-spherocytic hemolytic anaemia and neuromuscular dysfunction. Patients with inherited GPI deficiency show reduction in the synthesis of glycerolipids. Patients with rheumatoid arthritis have increased serum levels of GPI [41].

Fructose 6-phosphate may either be combined with glutamine to initiate the hexosamine pathway or continue the glycolytic pathway to form pyruvate that may be decarboxylated into acetyl-CoA to initiate fatty acid synthesis.

##### Hexosamine pathway

**Figure 5:**
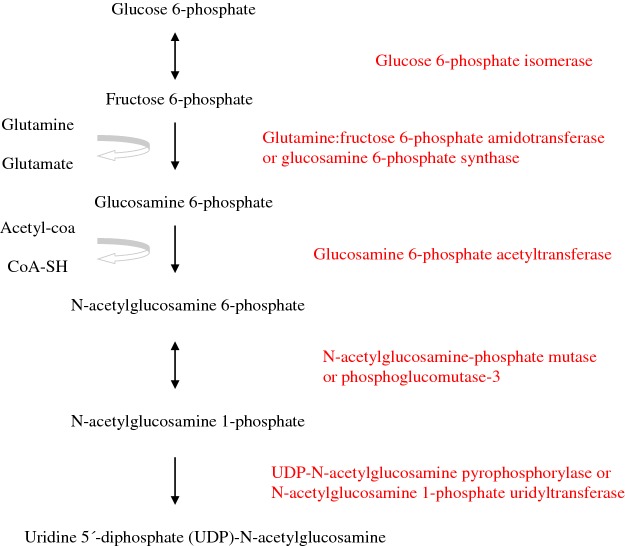
Hexosamine pathway

The hexosamine pathway produces UDP-*N*-acetylglucosamine and glutamate from glucose and glutamine (Figure 5). UDP-*N*-acetylglucosamine yields *N*-acetylglucosamine residues to build glycans that are attached to proteins and lipids, similarly to other nucleotide sugars such as UDP–galactose, being an essential precursor for glycosylation reactions. The enzyme that catalyses the addition of *N*-acetylglucosamine moieties to proteins is UDP-*N*-acetylglucosamine transferase whereas the enzyme that catalyses the removal of *N*-acetylglucosamine units from proteins is *N*-acetylglucosaminidase [42]. *In vitro* studies using HeLa and HEK293T cell lines show that *N*-acetylglucosamine O-glycosylation by human UDP-*N*-acetylglucosamine transferase may indirectly regulate DNA demethylation, although the physiological implication of this finding is uncertain. The extent of *N*-acetylglucosamine glycosylation is regulated by the concentration of glucose in the culture medium. High levels of glucose lead to increased *N*-acetylglucosamine glycosylation of proteins, presumably as a result of increased production of intracellular UDP-*N*-acetylglucosamine [43].

The sequence of reactions that composes the hexosamine pathway to synthesize UDP-*N*-acetylglucosamine in humans starts with the formation of fructose 6-phosphate from glucose 6-phosphate catalysed by GPI. Next, the enzyme glucosamine 6-phosphate synthase or glutamine:fructose 6-phosphate amidotransferase (GFAT) generates glucosamine 6-phosphate from fructose 6-phosphate and L-glutamine. Acetylation of glucosamine 6-phosphate by glucosamine 6-phosphate *N*-acetyltransferase (GNA1) renders *N*-acetylglucosamine 6-phosphate. *N*-acetylglucosamine-phosphate mutase (AGM1), also named PGM3, catalyses the reversible interconversion between *N*-acetylglucosamine 6-phosphate and *N*-acetylglucosamine 1-phosphate. Finally, the enzyme UDP-*N*-acetylglucosamine pyrophosphorylase (UAP1) or *N*-acetylglucosamine 1-phosphate uridyltransferase converts *N*-acetylglucosamine 1-phosphate into UDP-*N*-acetylglucosamine, the end product of the hexosamine pathway [40].

###### Synthesis of glucosamine 6-phosphate

Glucosamine 6-phosphate synthase or GFAT catalyses the formation of glucosamine 6-phosphate + glutamate from fructose 6-phosphate + glutamine. Glucosamine 6-phosphate synthase transfers the amino group from the L-glutamine amide to D-fructose 6-phosphate producing glutamate and glucosamine 6-phosphate. Human glucosamine 6-phosphate synthase has been cloned and the functional protein has been expressed in *Escherichia coli*. The gene coding human glucosamine 6-phosphate synthase has been mapped to chromosome 2p13. The end product of the hexosamine pathway, UDP-*N*-acetylglucosamine, exerts a feedback inhibition upon glucosamine 6-phosphate synthase activity. *In vitro* studies show that saturated fatty acids palmitate (C16:0) and stearate (C18:0) increase the expression of glucosamine 6-phosphate synthase mRNA and protein in cultured myotubes from human skeletal muscle whereas unsaturated fatty acids or glucose have no effect [44].

###### Acetylation of glucosamine 6-phosphate into *N*-acetylglucosamine 6-phosphate

The enzyme GNA1 catalyses the transfer of an acetyl group from acetyl-CoA to the primary amine of glucosamine 6-phosphate producing *N*-acetylglucosamine 6-phosphate and free coenzyme A (CoA-SH). The crystal structure of this enzyme from human liver has been ascertained. The human gene coding *GNA1* is located to 14q22.1 [45]. Human *GNA1* is able to transfer to glucosamine 6-phosphate acyl groups up to four carbons in length, including acetyl, propionyl, butyryl and isobutyryl whereas isovaleryl-CoA and decanoyl-CoA do not serve as donor substrates [46]. Glucose 6-phosphate inhibits human *GNA1* and the binding site of glucose 6-phosphate has been identified [47].

###### Formation of *N*-acetylglucosamine 1-phosphate from *N*-acetylglucosamine 6-phosphate

In 2002, it was noticed that the human enzyme AGM1 is identical with PGM-3. This enzyme is encoded by the gene *AGM1* and catalyses the reversible interconversion of *N*-acetylglucosamine 6-phosphate and *N*-acetylglucosamine 1-phosphate (Figure 2). The gene *AGM1* maps to chromosome 6 [24].

Autosomal recessive mutations in the *AGM1* gene cause CDG characterized by wide clinical manifestations including severe atopy, increased serum IgE levels, immune deficiency, autoimmunity and neurocognitive impairment from early life. Atopic diatheses may include asthma and food, drug and environmental allergies. Defects in T-cell function are suggested by persistent low-level EBV viraemia despite detectable EBV IgG and the development of EBV nodular sclerosing Hodgkin lymphomas. The patients may have recurrent skin and soft tissues infections, otitis, pulmonary infections, bronchiectasies and chronic respiratory failure. Immune-mediated disease may develop, including cutaneous leukocytoclastic vasculitis and membranoproliferative glomerulonephritis. In these patients, impaired function of PGM-3 is demonstrated by decreased enzyme activity and reduced UDP-*N*-acetylglucosamine, along with decreased O- and N-linked protein glycosylation. *N*-acetylglucosamine supplementation restores intracellular UDP-*N*-acetylglucosamine levels in PGM-3-deficient cells [48].

Homozygous mutations in *PGM3* have been identified to cause a type of primary immunodeficiency termed congenital hyper-IgE syndrome, associated with impaired T-cell function [49].

The pathophysiological mechanisms linking PGM-3 deficiency with CDG and primary immunodeficiency syndromes are unclear. Accurate glycosylation of immune receptors, immunoglobulins, proteins of complement and cytokines may be essential for the integrity of immune function [48].

###### Formation of UDP-*N*-acetylglucosamine from *N*-acetylglucosamine 1-phosphate

**Figure 6:**
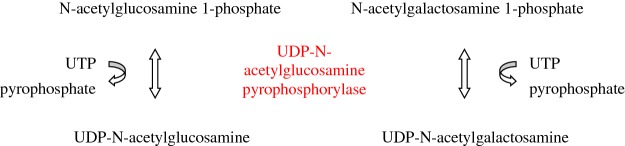
UDP-N-acetylglucosamine pyrophosphorylase reactions

*N*-acetylglucosamine 1-phosphate is converted into UDP-*N*-acetylglucosamine, the end product of the hexosamine pathway, by the enzyme UAP1 or *N*-acetylglucosamine 1-phosphate uridyltransferase. Human UAP1 is encoded by the gene *UAP1* located to 1q23.3. The human UAP1 cDNA isolated from a human testis cDNA library is identical with previously reported AGX1. UAP1 is highly expressed in prostate cancer and confers cancer cells growth advantage. UAP1 catalyses the reversible transfer of an uridyl group from UTP to *N*-acetylglucosamine 1-phosphate in the presence of Mg^2+^ or Mn^2+^, producing UDP-*N*-acetylglucosamine and releasing PP_i_ from UTP [50]. UAP1 also catalyses the synthesis of UDP-*N*-acetylgalactosamine from *N*-acetylgalactosamine 1-phosphate and UTP (Figure 6). The crystal structure of UAP1 has been solved [50].

##### Glucose oxidation to carbon dioxide

But for the post-prandial period, the liver is not predominantly oxidative, unlike the brain and the active skeletal muscle that consume most of the circulating glucose. Glucose is oxidized to carbon dioxide through a series of metabolic pathways, namely glycolysis in the cytosol followed by the tricarboxylic acid (TCA) cycle and the respiratory chain in the mitochondrial network. Glycolysis produces a small amount of ATP, but most ATP is generated through the oxidative phosphorylation of ADP, an oxygen-consuming reaction that takes place in the mitochondrial network.

The glycolytic pathway produces pyruvate from glucose in the cytosol. A small amount of ATP and NADH is generated (2 mol of ATP and NADH per mol of glucose). Oxygen is not required for glycolysis to proceed. Glucose is sequentially converted into glucose 6-phosphate, fructose 6-phosphate and fructose 1,6-bisphosphate, which is split into dihydroxyacetone phosphate and glyceraldehyde 3-phosphate, two trioses that may be converted into each other. Glyceraldehyde 3-phosphate is sequentially transformed into 1,3-bisphosphoglycerate, 3-phosphoglycerate, 2-phosphoglycerate, phosphoenolpyruvate and pyruvate [51].

Pyruvate dehydrogenase (PDH) is a multiprotein complex that catalyses the irreversible oxidative decarboxylation of pyruvate to acetyl-CoA in the mitochondrial network, whereas NAD^+^ is reduced to NADH. The PDH reaction allows the entrance of acetate into the TCA cycle. Congenital PDH deficiency usually presents during the first year of life and is characterized by heterogeneous neurological features, hyperammonaemia and lactic acidosis [52].

Acetate enters the TCA cycle as acetyl-CoA, being combined with oxaloacetate to form citrate whereas CoA is liberated. Citrate is sequentially converted into isocitrate, 2-oxoglutarate (α-oxoglutarate), succinyl-CoA, succinate, fumarate, L-malate and finally oxaloacetate, closing the cycle. Several reactions in the TCA cycle provide NADH and FADH_2_ that are subsequently oxidized in the mitochondrial respiratory chain to produce ATP [53].

NADH and FADH_2_ generated during glycolysis and the TCA cycle are oxidized to NAD^+^ and FAD in the inner mitochondrial membrane providing reducing equivalents (electrons) that are transported along the components of the respiratory chain to reach ultimately molecular oxygen, which is reduced to water. The transfer of reducing equivalents through the components of the respiratory chain supplies the energy that is used to synthesize ATP via the oxidative phosphorylation of ADP [54].

##### Fatty acid synthesis from acetyl-CoA

Surplus dietary carbohydrate stimulates whole body carbohydrate oxidation while suppressing the oxidation of fat. However, the capacity to oxidize excess dietary carbohydrate is limited and when surpassed, additional glucose is converted into fatty acids in the liver. Excess dietary carbohydrate increases body fat stores both by suppression of the oxidation of dietary fat and by conversion of the surplus carbohydrate to fat [35].

Among healthy subjects, carbohydrate overfeeding increases hepatic *de novo* lipogenesis compared with a control diet. Glucose and sucrose have similar effect increasing *de novo* lipogenesis when overfed. After glucose loading, lipogenesis is markedly increased at the expense of glycogen synthesis. Further, net *de novo* lipogenesis from carbohydrate occurs in normal volunteers who are in calorie balance, as the consumption of a eucaloric low fat high carbohydrate diet increases palmitate synthesis and elevates the plasma concentration of palmitate-enriched triacylglycerol in VLDL particles. Conversely, low carbohydrate diets reduce *de novo* lipogenesis [35].

In the cytosol of hepatocytes, fatty acids are synthesized from acetyl-CoA exported from the mitochondria. NADPH derived from the pentose phosphate pathway is required for the reductive synthesis of fatty acids. Acetyl-CoA formed inside the mitochondria may be exported to the cytosol either as acetyl-carnitine generated by the enzyme carnitine acetyltransferase (CRAT) or as citrate formed in the first reaction of the TCA cycle. CRAT catalyses the reversible transfer of short-chain acyl groups such as acetyl-CoA between the CoA and L-carnitine in the mitochondrial matrix of hepatocytes. The formation of acetyl-carnitine allows the exit of acetyl groups from the mitochondria into the cytoplasm [55]. In the first reaction of the TCA cycle, acetyl-CoA is combined with oxaloacetate by the enzyme citrate synthase generating citrate. Mitochondrial citrate is exported to the cytoplasm where the citrate cleavage enzyme or ATP citrate lyase (ACLY) reforms acetyl-CoA and oxaloacetate from citrate. ACLY is acetylated at lysine residues. Acetylation increases enzyme stability. Conversely, the protein deacetylase sirtuin-2 deacetylates and destabilizes ACLY [56].

The enzyme acetyl-CoA carboxylase catalyses the carboxylation of acetyl-CoA into malonyl-CoA. Acetyl-CoA and malonyl-CoA are utilized to synthesize long-chain fatty acids, such as palmitate. Insulin and citrate activate acetyl-CoA carboxylase, promoting malonyl-CoA formation and the synthesis of fatty acids [57]. In contrast, glucagon and palmitate inhibit acetyl-CoA carboxylase and the synthesis of fatty acids. Phosphorylation of acetyl-CoA carboxylase by 5′-AMP-dependent protein kinase (AMPK) inactivates the enzyme, inhibiting malonyl-CoA formation and the synthesis of fatty acids [57].

Cytosolic fatty acid synthase catalyses the *de novo* synthesis of long-chain fatty acids from acetyl-CoA, malonyl-CoA and NADPH. Palmitate or hexadecanoate (C16:0), a 16-carbon saturated fatty acid, is the main product of the reaction catalysed by fatty acid synthase in the cytosol. Human fatty acid synthase-1 is a homodimer. Each of the two identical dimers possesses seven structural and functional domains [58].

#### Pentose phosphate pathway

**Figure 7:**
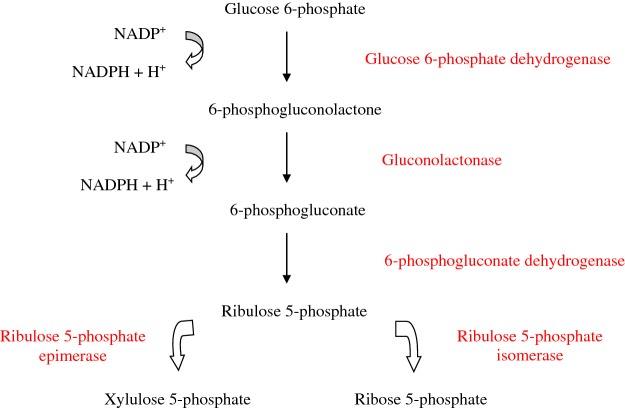
Pentose phosphate pathway

The pentose phosphate pathway is a physiological route of glucose metabolism in the cytosol that provides reducing equivalents (NADPH) and ribose 5-phosphate (Figure 7). In the hepatocytes, NADPH is required for the synthesis of fatty acids. In the red blood cells, NADPH is predominantly used to maintain glutathione in the reduced state, protecting cells from oxidative damage. Ribose 5-phosphate is a pentose required for the synthesis of nucleotides, such as those found in RNA, DNA, NADH, FAD or CoA.

In the first step of the pentose phosphate pathway, glucose 6-phosphate is oxidized into gluconolactone and carbon dioxide by glucose 6-phosphate dehydrogenase whereas NADP^+^ is reduced to NADPH. Studies *in vitro* have shown that glucose 6-phosphate dehydrogenase undergoes *O*-linked β-*N*-acetylglucosamine glycosylation in response to hypoxia. Glycosylation activates the enzyme and increases glucose flux through the pentose phosphate pathway, but the clinical implication of this finding is uncertain. In the second reaction of the pentose phosphate pathway, gluconolactonase converts gluconolactone into 6-phosphogluconate. Then, 6-phosphogluconate is oxidized by 6-phosphogluconate dehydrogenase to yield ribulose 5-phosphate whereas NADP^+^ is reduced to NADPH. Ribulose 5-phosphate may either be isomerized to ribose 5-phosphate or epimerized into xylulose 5-phosphate. Transketolase is responsible for the cleaving of a two-carbon unit from xylulose 5-phosphate and adding that two carbon unit to ribose 5-phosphate resulting in glyceraldehyde 3-phosphate and sedoheptulose 7-phosphate. The combination of glyceraldehyde 3-phosphate and dihydroxyacetone phosphate by aldolase produces fructose 6-phosphate. Transaldolase is responsible for cleaving a three carbon unit from sedoheptulose 7-phosphate and adding that three carbon unit to glyceraldehyde 3-phosphate resulting in erythrose 4-phosphate and fructose 6-phosphate. Transketolase may also be responsible for the cleaving of a two carbon unit from xylulose 5-phosphate and adding that two carbon unit to erythrose 4-phosphate resulting in glyceraldehyde 3-phosphate and fructose 6-phosphate [59].

Glyceraldehyde 3-phosphate and fructose 6-phosphate are intermediates of the glycolytic pathway and the pentose phosphate pathway, connecting the two metabolic routes. Rapidly dividing cells such as cancer cells require activation of the pentose phosphate pathway to produce DNA for rapid cellular division, explaining both the excessive consumption of glucose in the presence of oxygen by fast-growing tumour cells (Warburg effect) and the excessive production of lactate associated with uncontrolled malignancy. Congenital deficiency involving enzymes of the pentose phosphate pathway has been rarely reported, except for the first enzyme, glucose 6-phosphate dehydrogenase. Congenital deficiency of glucose 6-phosphate dehydrogenase is a common disease that causes hemolytic anaemia and neonatal ictericia [60].

## HEPATIC GLUCOSE PRODUCTION

The human liver possesses the remarkable ability to produce glucose that is released to the systemic circulation and used by other tissues, particularly during periods of fasting. Hepatic glucose production derives from glycogen breakdown (glycogenolysis) and from *de novo* synthesis of glucose (gluconeogenesis). The liver is the main human tissue able to synthesize glucose although the proximal tubule of the kidney may produce a limited amount from carbohydrate skeletons of amino acids used to produce ammonium, such as glutamine, particularly in response to acidosis.

Both gluconeogenesis and glycogenolysis contribute to hepatic glucose production. During short-term periods of fasting, glycogenolysis is the predominant source of glucose released to the bloodstream. However, during prolonged periods of fasting, the glycogen reserve is gradually consumed and glycogenolysis decreases as glycogen store is depleted. Then, gluconeogenesis becomes the predominant source of glucose to the human body. The contribution of gluconeogenesis to hepatic glucose production increases gradually with prolonged fasting so that after approximately 42 h of fasting, gluconeogenesis accounts for almost all of glucose production in healthy subjects. Likewise, during insulin-induced hypoglycaemia (55 mg/dl) that mimics starvation periods in healthy volunteers, glycogenolysis accounts initially for 85% of hepatic glucose output, but once hypoglycaemia becomes established the contribution of gluconeogenesis increases to 77–94% [61].

Among healthy individuals, a reduction of fatty acid availability inhibits gluconeogenesis, the rate of gluconeogenesis being positively correlated with the rate of fatty acid oxidation. Reduced fatty acid oxidation in the liver suppresses gluconeogenesis due at least in part to decreased production of acetyl-CoA, which is an activator of pyruvate carboxylase [4,62]. Insulin inhibits adipose lipolysis and consequently reduces plasma concentration of fatty acids and fatty acid availability to be oxidized, suppressing gluconeogenesis. However, the inhibitory effect of insulin on hepatic gluconeogenesis is limited despite its suppressing effect on adipose lipolysis. Insulin infusion to normal subjects fasted overnight almost completely suppresses fatty acid availability and oxidation, but gluconeogenesis flux is reduced by only 20%. In healthy subjects, insulin reduces hepatic glucose output predominantly by reducing glycogenolysis and enhancing glycogen accumulation [4]. By contrast, glucagon has a transient effect reducing liver glycogen content and raising plasma glucose concentration. Net hepatic glycogenolysis accounts for 93% of the increase in hepatic glucose production during glucagon infusion to healthy volunteers. The transitory effect of glucagon on hepatic glucose production is not caused by depletion of hepatic glycogen stores [63].

After ingestion of a mixed meal, hepatic glucose output is inhibited predominantly at the expense of a suppression of glycogenolysis. Gluconeogenesis remains active during the post-prandial period, although its rate is attenuated [33]. The suppression of glycogenolysis and to a lesser extent gluconeogenesis and the activation of glycogen synthesis during the post-prandial period is mainly driven by stimulation of insulin secretion and suppression of glucagon secretion [64].

### Gluconeogenesis

The liver synthesizes glucose *de novo* from precursors such as fructose, lactate, alanine and glycerol via the gluconeogenesis pathway. Glutamine is a predominant precursor of gluconeogenesis in the kidney. Glucose synthesized from gluconeogenesis in the liver is used to replenish liver glycogen stores and to supply glucose to the bloodstream.

Aldolase B catalyses the conversion of fructose 1-phosphate into dihydroxyacetone phosphate and glyceraldehyde 3-phosphate, two trioses that may combine to form fructose 1,6-bisphosphate, which is incorporated to the gluconeogenesis pathway to produce glucose. Congenital deficiency of aldolase B causes hereditary fructose intolerance [65]. Fructose 1,6-bisphosphatase catalyses the dephosphorylation of fructose 1,6-bisphosphate to fructose 6-phosphate and P_i_ in the cytosol. Congenital deficiency of fructose 1,6-bisphosphatase is a rare autosomal recessive disorder leads to impaired gluconeogenesis. Affected patients usually present with episodes of severe hyperventilation due to lactic acidosis and hypoglycaemia occurring with fasting [66].

Lactate dehydrogenase (LDH) catalyses the reversible interconversion of lactate and pyruvate. Oxidation of lactate yields pyruvate whereas NAD^+^ is reduced to NADH. Reduction of pyruvate by LDH renders lactate whereas NADH is oxidized to NAD^+^. In the skeletal muscle, the LDH reaction proceeds towards lactate formation. Lactate is then transported to the liver where LDH acts in the opposite direction, generating pyruvate to produce glucose via the gluconeogenesis pathway [67].

Alanine aminotransferase (ALT) or glutamate pyruvate transaminase catalyses the reversible transamination of L-alanine and α-oxoglutarate (2-oxoglutarate) to yield pyruvate and L-glutamate respectively. Similarly to lactate, the reaction proceeds predominantly towards the formation of alanine in the skeletal muscle. Alanine is then transported to the liver to form pyruvate that is used to synthesize glucose by the gluconeogenesis pathway. Among healthy subjects, alanine is the principal amino acid released by skeletal muscle and extracted by the liver to generate glucose during the post-absorptive period. In healthy subjects fasted for 60 h, alanine infusion is accompanied by an 80% rise in splanchnic glucose output and a 16% rise in arterial glucose concentration. Likewise, an increment in blood glucose level is demonstrable after alanine administration in subjects fasted 3–4 weeks [68].

Glycerol contribution to endogenous glucose production ranges from approximately 3%–22% depending on the duration of fasting. In the post-absorptive state, approximately 3% of plasma glucose is derived from glycerol, but glycerol contributes approximately 22% of endogenous glucose production with prolonged fasting among healthy humans. Under this condition, up to 100% of the glycerol turnover is diverted to glucose formation [69].

To produce glucose in the liver, lactate and alanine are first converted to pyruvate. The carboxylation of pyruvate into oxaloacetate by pyruvate carboxylase is the first reaction of the gluconeogenic pathway from lactate and alanine and occurs inside the mitochondrial network. Acetyl-CoA is the allosteric activator of human pyruvate carboxylase and therefore accumulation of acetyl-CoA from fatty acid oxidation or other sources stimulates gluconeogenesis. During fasting, pyruvate carboxylation in the liver is activated to produce glucose whereas pyruvate decarboxylation by the PDH complex to oxidize glucose is suppressed [62]. The enzyme phosphoenolpyruvate carboxykinase (PEPCK) catalyzes the formation of phosphoenolpyruvate from oxaloacetate. In the cytosol, phosphoenolpyruvate is sequentially transformed into 2-phosphoglycerate, 3-phosphoglycerate, 1,3-bisphosphoglycerate, and glyceraldehyde 3-phosphate, a triose that may be interconverted into dihydroxyacetone phosphate. The combination of glyceraldehyde 3-phosphate and dihydroxyacetone phosphate produces fructose 1,6-bisphosphate. The dephosphorylation of fructose 1,6-bisphosphate renders fructose 6-phosphate, which is transformed into glucose 6-phosphate [70]. The enzymes pyruvate carboxylase, PEPCK and fructose 1,6-bisphosphatase catalyse irreversible steps in the gluconeogenesis pathway. Congenital deficiency of any of these enzymes leads to intolerance to fasting with ketotic hypoglycaemia and lactic acidosis.

Unlike lactate and alanine that are converted to pyruvate and then into oxaloacetate and phosphoenolpyruvate to synthesize glucose, glycerol derived from triacylglycerols is incorporated to the gluconeogenesis pathway by being converted into dihydroxyacetone phosphate, producing glucose avoiding phosphoenolpyruvate formation [69].

### Glycogenolysis

After meals, glucose is stored as glycogen in the liver. During fasting periods, glucose is released from glycogen (glycogenolysis) becoming available to be used in other tissues. Liver glycogen content falls overnight to its daily minimum value before breakfast. Glycogen degradation in the cytosol of hepatocytes is accomplished by two enzymes. Glycogen phosphorylase releases glucose 1-phosphate from the linear chain of glycogen whereas glycogen debranching enzyme unfastens the branch points of the glycogen particle. Congenital deficiency of hepatic glycogen phosphorylase (glycogen storage disease type VI or Hers disease) leads to reduced ability to mobilize glucose from glycogen in response to fasting and glucagon [71]. Congenital deficiency of glycogen debranching enzyme (glycogen storage disease type III or Cori–Forbes disease) results in accumulation of abnormal glycogen in affected tissues, including liver, heart and skeletal muscle. Liver involvement is characterized by hepatomegaly, hepatic fibrosis and hepatic adenomata. Intolerance to fasting with hypoglycaemia also occurs. Hypertriglyceridaemia and hypercholesterolaemia are common in patients with congenital deficiency of glycogen debranching enzyme. Elevated plasma concentration of medium-chain fatty acids, predominantly C8 and C10, has been reported in one patient with this disease [72].

### Glucose dephosphorylation

In order to leave the hepatocyte, glucose 6-phosphate derived from either gluconeogenesis or glycogenolysis is dephosphorylated to free glucose in the endoplasmic reticulum.

Glucose 6-phosphate translocase transports glucose 6-phosphate from the cytosol into the lumen of the endoplasmic reticulum and glucose 6-phosphatase isoenzymes catalyse the dephosphorylation of glucose 6-phosphate to yield free glucose and P_i_ [73].

Congenital deficiency of either glucose 6-phosphate translocase or glucose 6-phosphatase isoforms causes glycogen storage disease type 1 (von Gierke disease). The release of free glucose from the hepatocyte to the bloodstream is impaired leading to hypoglycaemia during fasting periods. The intracellular concentration of glucose 6-phosphate increases promoting alternative pathways to use glucose, including accumulation of glycogen and excess formation of fatty acids, lactic acid and uric acid [74]. Hepatic *de novo* lipogenesis is markedly enhanced in patients with glycogen storage disease type 1 as revealed by an increased contribution of newly synthesized palmitate in VLDL compared with controls. The lipid profile of these patients is characterized by increased plasma concentration of triacylglycerol and cholesterol. Dietary management to promote normal glucose levels improves plasma level of triacylglycerol but does not usually normalize it [75].

In addition to this metabolic phenotype, some patients with glucose 6-phosphate translocase deficiency develop neutropenia and neutrophil dysfunction with tendency to infections [76].

Patients with congenital deficiency of glucose 6-phosphate translocase or glucose 6-phosphatase-C3 exhibit a severe defect in the synthesis of N- and O-glycans in the neutrophils. The mechanism of this anomalous glycosylation is unclear, but it is predicted to have a major negative effect on neutrophil function. It has been proposed that both severe congenital neutropenia type 4 (due to mutations in the glucose 6-phosphatase-C3 gene) and glycogenosis type Ib (due to mutations in the glucose 6-phosphate translocase gene) should be designated a new CDG [77].

### Glucose transport outside the hepatocyte: glucose transporter-2

The mechanisms involved in glucose exit from the hepatocytes to the bloodstream have not been investigated. Patients harbouring mutations in the *SLC2A2* gene (Fanconi–Bickel disease) suffer fasting hypoglycaemia and liver glycogen accumulation, indicating that GLUT2 is required for glucose to leave the hepatocyte.

## SUMMARY

**Figure 8:**
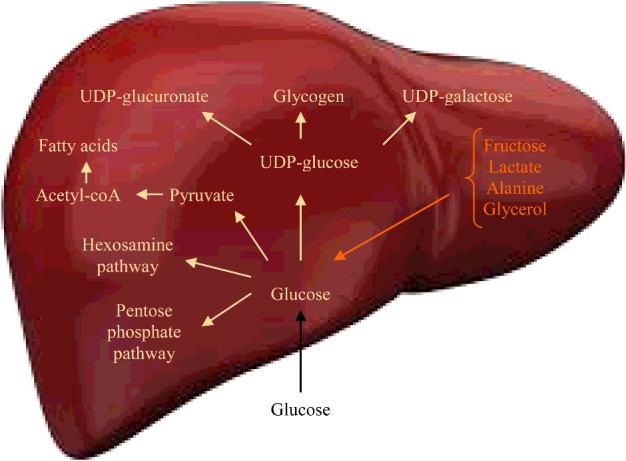
Integrative figure of liver glucose metabolism

Glucose reaches hepatic cells from the intestine via the portal vein and from the systemic circulation via the hepatic artery. Glucose entrance into human hepatocytes likely occurs without energy requirement through transporters. Inside the hepatocytes, free glucose is phosphorylated to glucose 6-phosphate by glucokinase. Glucose 6-phosphate may be utilized in a number of metabolic routes, including production of UDP–glucose and fructose 6-phosphate, and oxidation to initiate the pentose phosphate pathway. UDP–glucose is used to synthesize glycogen, UDP–glucuronate and UDP-galactose. Quantitatively, the most important pathway of glucose utilization in the liver is glycogen synthesis. Most glucose entering the hepatocyte after meals is stored as glycogen to create a reserve of fuel that can be used during fasting periods. Liver glucose may be utilized in other metabolic pathways that have important functions. UDP–glucuronate provides glucuronate residues to be attached to endogenous and exogenous compounds facilitating their excretion. UDP–glucuronate is used for glycosaminoglycan assembly as well. UDP–galactose participates in glycosylation of proteins and lipids. Fructose 6-phosphate may either be combined with glutamine to initiate the hexosamine pathway or continue the glycolytic pathway to produce pyruvate. The hexosamine pathway produces carbohydrate units used for glycosylation reactions. Excess dietary glucose is converted into fatty acids in the liver, increasing the amount of fat both in the liver and in the adipose tissue. Glucose 6-phosphate may be oxidized to initiate the pentose phosphate pathway, an important physiological route of glucose metabolism that provides NADPH and ribose 5-phosphate. NADPH is required for the synthesis of fatty acids whereas ribose 5-phosphate is needed for the synthesis of nucleotides, including those present in DNA and RNA. A vital function of the human liver is to make glucose available to other tissues such as the brain and the exercising skeletal muscle during periods of fasting or exercise (Figure 8).

Hepatic glucose metabolism has an important role in glycosylation processes of proteins and lipids that has not been fully elucidated. PGM-1 catalyses the reversible interconversion of glucose 1-phosphate and glucose 6-phosphate. PGM-3 catalyses a similar interconversion between *N*-acetylglucosamine 6-phosphate and *N*-acetylglucosamine 1-phosphate. Congenital deficiency of both PGM-1 and PGM-3 are associated with glycosylation disorders. Abnormal glycosylation of proteins leads to immune deficiency via unknown pathogenic mechanisms.

Mutations in the glucokinase gene cause diabetes mellitus, highlighting the role of the liver in the pathogenesis of this disease. Hepatic glycogen synthesis from dietary glucose is impaired in glucokinase-deficient patients. The role of hepatic glucose metabolism in the formation of the extracellular matrix including that of blood vessels wall via UDP–glucuronate generation and glycosaminoglycan construction has not been explored.

## Abbreviations

ACLY: ATP citrate lyase
AGM1: *N*-acetylglucosamine-phosphate mutase
CDG: congenital disorders of glycosylation
CRAT: carnitine acetyltransferase
GALE: UDP–galactose 4-epimerase
GALT: galactose 1-phosphate uridyltransferase
GFAT: glutamine:fructose 6-phosphate amidotransferase
GNA1: glucosamine 6-phosphate *N*-acetyltransferase
GPI: glucose 6-phosphate isomerase
HNF: hepatocyte nuclear factor
LDH: lactate dehydrogenase
MODY: maturity-onset diabetes of youth
PDH: pyruvate dehydrogenase
PEPCK: phosphoenolpyruvate carboxykinase
PGM: phosphoglucomutase
TCA: tricarboxylic acid
UAP1: UDP-*N*-acetylglucosamine pyrophosphorylase
UGP: UDP–glucose pyrophosphorylase
